# Changes in Laminin Expression Pattern during Early Differentiation of Human Embryonic Stem Cells

**DOI:** 10.1371/journal.pone.0138346

**Published:** 2015-09-17

**Authors:** Martin Pook, Indrek Teino, Ade Kallas, Toivo Maimets, Sulev Ingerpuu, Viljar Jaks

**Affiliations:** 1 Department of Cell Biology, Institute of Molecular and Cell Biology, University of Tartu, Tartu, Riia 23, 51010, Estonia; 2 Department of Biosciences and Nutrition, Karolinska Institutet, SE-141 83, Huddinge, Sweden; Johns Hopkins School of Medicine, UNITED STATES

## Abstract

Laminin isoforms laminin-511 and -521 are expressed by human embryonic stem cells (hESC) and can be used as a growth matrix to culture these cells under pluripotent conditions. However, the expression of these laminins during the induction of hESC differentiation has not been studied in detail. Furthermore, the data regarding the expression pattern of laminin chains in differentiating hESC is scarce. In the current study we aimed to fill this gap and investigated the potential changes in laminin expression during early hESC differentiation induced by retinoic acid (RA). We found that laminin-511 but not -521 accumulates in the committed cells during early steps of hESC differentiation. We also performed a comprehensive analysis of the laminin chain repertoire and found that pluripotent hESC express a more diverse range of laminin chains than shown previously. In particular, we provide the evidence that in addition to α1, α5, β1, β2 and γ1 chains, hESC express α2, α3, β3, γ2 and γ3 chain proteins and mRNA. Additionally, we found that a variant of laminin α3 chain—145 kDa—accumulated in RA-treated hESC showing that these cells produce prevalently specifically modified version of α3 chain in early phase of differentiation.

## Introduction

Human embryonic stem cells (hESC) are derived from the inner cell mass of blastocyst. They have the capacity to self-renew and differentiate into cells of all three embryonic germ layers [1]. Transcription factors OCT4, NANOG and SOX2 are important regulators for hESC to retain their pluripotency and self-renewing characteristics [2]. Both down and up regulation of the expression levels of these transcription factors can induce differentiation of hESC [3–6]. In a murine embryonic carcinoma cell line, retinoic acid (RA) has been shown to repress the expression of OCT4 via RAREs (retinoic acid response elements) present in the OCT4 promoter [7]. In monolayer hESC cell cultures, this chemical can induce neuronal [8,9] and endodermal differentiation [9] but can be used also to direct hESC towards extraembryonic lineages when specific conditions are provided [10]. If applied to differentiating cells from embryoid bodies, it can induce also differentiation towards mesodermal lineage [11].

The extracellular matrix (ECM), which surrounds cells both *in vivo* and in culture conditions, is essential in regulating stem cell differentiation and survival [12–14]. Laminins are integral components of the basement membrane—a specific form of ECM—and play a critical role in early development by coordinating the differentiation process [15]. Laminins are hetero-trimeric proteins composed of α, β and γ chains, which form at least 16 different isoforms that are named according to their chain composition (e.g. laminin-111 contains one α1, one β1 and one γ1 chain) [16].

The crosstalk between ECM and embryonic stem cells is complex by nature and is pivotal for regulating the balance between their differentiation and stemness [14]. The expression of single laminin β and γ chains can be detected already at 2-4-cell embryo stage [17,18] suggesting the importance of laminin in guiding the earliest steps of embryonic development. The earliest trimeric laminins expressed during mammalian embryogenesis are laminin-111 and -511 [15] and embryos lacking α1 [19] or α5 [20] chains die at an early developmental stage. It is now known that the pluripotent hESC cultured *in vitro* express laminin α1, α5, β1, β2 and γ1 chains [21,22] although some studies failed to detect the presence of β2 chain [23,24]. The importance of these laminin chains in the maintenance of hESC is further reinforced with the data that the cultivation of hESC on recombinant laminin-511 or -521 efficiently preserves the pluripotency of these cells [22,25].

The changes in the expression of different laminin chains at different developmental stages have been described in detail [26]. Less is known about the changes in laminin expression pattern during early steps of embryonic stem cell differentiation. Furthermore, despite of the fact that laminins 511 and 521 have distinct functions during mammalian development [27,28] the potential interplay between these laminin isoforms has not been addressed during the initiation of hESC differentiation.

In the current study we aimed to characterize the changes in laminin composition of the ECM produced by hESC during early differentiation induced by RA. We uncovered an intricate interplay between laminin-511 and -521 during early differentiation. Using immunoprecipitation of α5-laminins we found that the relative amount of laminin-511 is increased when compared to laminin-521 suggesting that the changes in the proportion of these two laminin isoforms contribute to the coordination of the early steps of hESC differentiation. Furthermore, we found that the laminin chain repertoire present in cultured hESC is more diverse than previously described. In addition to laminin α1, α5, β1, β2 and γ1 chains, we were able to detect the expression of α2, α3, β3, γ2 and γ3 chains at the mRNA and protein level.

## Materials and Methods

### Ethics statement

The permit to isolate mouse embryonic fibroblasts from the embryos of CD-1 mice (Animal Facility, Institute of Molecular and Cell Biology, Tartu, Estonia) was obtained from the Committee for Experiments on Laboratory Animals, Estonian Ministry of Agriculture. Mice were sacrificed by cervical dislocation.

### Cell culture

To maintain the human embryonic stem cells (WA09, National Stem Cell Bank) in pluripotent state the cells were cultured on 6-well tissue culture plates (BD Biosciences) coated with Matrigel (BD Biosciences) in mTeSR1 media (STEMCELL Technologies) according to the manufacturer’s specifications. Cells were passaged mechanically after 3–4 days. For cell passage, hESC colonies were detached with a micropipette tip and dissociated by gentle pipetting using serological pipette. Cells were cultured in 5% CO_2_ at 37° C in humid conditions. The culture medium was changed daily. The normal karyotype of the cells was confirmed by G-banding.

### Induction of hESC differentiation

Mouse embryonic fibroblast (MEF) feeder cells were derived from 12.5-day embryos (CD1) followed by irradiation with 7000 rad (x-ray irradiator RX-650, Faxitron Bioptics). The irradiated MEFs were cultured at density 4.24 x 10^4^ cells/cm^2^ in Dulbecco’s modified Eagle’s medium supplemented with 20% knockout serum replacement, 0.1 mM nonessential amino acids, 1 mM L-glutamine, and 4 ng/ml human basic fibroblast growth factor (all from Invitrogen); 0.0007% 2-mercaptoethanol (Sigma-Aldrich) for 24 hours to produce conditioned media termed hereafter "differentiation media". One day after passaging, the hESC differentiation was induced by replacing the mTeSR1 growth media with the differentiation media containing 10 μM retinoic acid (RA, Sigma-Aldrich) in DMSO. The final concentration of DMSO in the differentiation media was 0.1%. Subsequently, hESC were cultured for 5 days and differentiation media containing RA was replaced daily.

### Immunofluorescence microscopy

For immunofluorescence (IF) analysis, cells were cultured on glass coverslips (Marienfeld) coated with Matrigel. Cells were fixed with 4% paraformaldehyde in PBS for 15 min at room temperature. After 3x5 min washing with PBS, cells were permeabilized with 0.2% Triton X-100 in PBS at room temperature for 10 min. Subsequently, cells were washed with PBS for 3 times and blocked with 4% normal goat serum in PBS for 60 min at room temperature. Primary and secondary antibodies were diluted in 4% normal goat serum in PBS and all incubations were carried out in a humid chamber. Each staining was followed by washing the cells with PBS for 3x5 minutes. Cell nuclei were visualized by staining with DAPI (1 μg/mL, Sigma-Aldrich) for 15 min at room temperature. Slides were mounted in Dako Fluorescent Mounting Medium (Dako). The images were obtained with fluorescence microscope IX81 or confocal microscope FV1000 (both from Olympus Corporation) and analyzed with Imaris software (Bitplane AG). The antibodies used in the study are described in Supporting Information (Table A in S1 File). Three independent IF analyses were performed.

### Western blot analysis

Samples were collected by scraping the cells with rubber policeman in radioimmunoprecipitation assay (RIPA) buffer containing 10 mM Tris-HCl (pH 7.2), 150 mM NaCl, 0.1% SDS, 1.0% Triton X-100, 1% sodium deoxycholate, 5 mM EDTA and Complete protease inhibitor cocktail (Roche Diagnostics). The concentration of total protein was measured by using BCA Protein Assay Kit (Thermo Fisher Scientific) and equal protein amounts for each sample were loaded to SDS-PAGE gels for electrophoresis (Mini-Protean system, Bio-Rad Laboratories). For detection of laminin chains, 5% and for all other proteins of interest 10% gels were used. Transfer to polyvinylidene difluoride membrane was carried out using Mini Trans-Blot Cell system (Bio-Rad Laboratories). The membrane was washed with TBS-T (Tris-Buffered Saline, 0.1% Tween 20) and blocked with 5% skimmed milk solution in TBS-T (blocking solution) for 1 h followed by incubation with primary antibodies in blocking solution overnight at 4°C. After washings with TBS-T, the membrane was incubated with the respective secondary antibody conjugated with horseradish peroxidase (HRP) for 1 h at room temperature. Subsequently, the membrane was rinsed three times with TBS-T for 15 min followed by incubation with Immobilon Western Chemiluminescent HRP Substrate solution (Millipore Corporation). Chemiluminescent signal was detected and quantified using Biospectrum 510 Imaging System with VisionWorks LS software (both UVP, LLC) or by exposing to x-ray film (Agfa HealthCare NV). The antibodies used in the study are described in Supporting Information (Table A in S1 File).

As positive controls for laminin chain expression a selection of human cells and cell lines were used. JEG-3 cells were used to identify laminin α1 chain, A431 cells for detecting α2 and α3 chains while α4 chain was validated using lysate prepared from human platelets. The laminin α5 chain was identified using lysates prepared from A549, A431, JAR and JEG-3 cells. All other chains (β1-β3, γ1- γ3) were identified using the lysate prepared from A431 cells. Two independent Western blot analyses were performed.

### Immunoprecipitation assay

To immunoprecipitate the laminin isoforms, which contain α5 chain the cells were lysed by scraping the cells with rubber policeman in RIPA buffer and Complete protease inhibitor cocktail (Roche Diagnostics). First, 400 μL of magnetic beads, (Dynabeads M-280 coated with Sheep anti-Mouse IgG, 2.68 x 10^8^ beads, Thermo Fisher Scientific) and 24 μg (9.8 μL) of laminin α5 chain mouse monoclonal antibody 4B5 (Table A in S1 File) were mixed and incubated in a rotating tube for 3.5 h at 4°C. Next, 200 μL of the antibody-coupled bead suspension and 150 μL of cell lysate containing 128.2 μg of total protein were mixed and incubated in a rotating tube for 12h at 4°C. Beads were rinsed before and after incubations 3 times for 2 minutes using Ca 2^+^/ Mg 2^+^ −free PBS supplemented with 0.1% bovine serum albumin and 2 mM EDTA. To extract the bound proteins, the beads were suspended in electrophoresis sample buffer containing SDS, dithiothreitol (DTT) and incubated at 100°C for 5 min. The supernatant was used to perform the Western blot analysis as described in the previous section. Antibodies recognizing laminin chains β1-β3 and γ1- γ3 (Table A in S1 File) were used to detect the α5-bound laminin subunits in a subsequent Western blot analysis. The beads were extracted from the suspension after each incubation or washing step using a dedicated magnetic rack (Dynal). The summarized results of quantification are included in the Supporting Information (Table B in S1 File). To evaluate the gross amount of laminin α5 chain, the corresponding signal values were normalized to the actin signal values of the corresponding input (Table B in S1 File). The amount of each laminin chain bound to the α5 chain was calculated by normalization of the signal values corresponding to β1, β2 and γ1 chains to the corresponding α5 chain signal intensity values (Table B in S1 File). Two independent immunoprecipitation experiments were conducted.

### Reverse transcription PCR (RT-PCR)

RNA samples were collected from hESC treated with RA using Trizol Reagent (Invitrogen). RNA was isolated with miRNeasy kit (Qiagen) followed by treatment with DNase I (Thermo Fisher Scientific). RevertAid First Strand cDNA Synthesis Kit (Thermo Fisher Scientific) with random hexamer primers was used to synthesize cDNA. RT-PCR was performed using FirePol Master Mix (Solis BioDyne) and specific primers as described in Supporting Information (Table C in S1 File). PCR products were separated using 2% agarose gel electrophoresis and images were obtained using Biospectrum 510 Imaging System (UVP, LLC). Two independent RT-PCR analyses were conducted.

### Multivariate permeabilized-cell flow cytometry

Cells treated with RA or DMSO were harvested with 0.05% trypsin-EDTA solution (PAA Laboratories) and washed with PBS. The single cell suspensions were fixed using 1.6% paraformaldehyde (PFA, Sigma-Aldrich) for 10 min at room temperature (RT). Cells were washed with permeabilization buffer (Permeabilization buffer, e-Biosciences), blocked using 2% goat serum (PAA Laboratories) in permeabilization buffer (10 min at RT) and stained with appropriate antibodies or their isotype controls (Table A in S1 File) for 30 min at RT. For cell cycle analysis cells were further stained with DAPI (Sigma-Aldrich). Compensation controls were prepared by using CompBead Plus compensation particles (BD Biosciences) incubated with appropriate antibodies. Flow cytometry data were acquired with FACSAria using FACSDiva software (BD Biosciences) and analyzed with FACSDiva (BD Biosciences) or FlowJo software (FlowJo, LLC). At least three independent flow cytometry experiments were conducted for each antibody combination except for detection of α1, α2 and α3 laminin chains where two independent experiments were performed.

### Statistical analysis

To analyze the data from flow cytometric analysis fold change between the Median Fluorescence Intensity (MFI) values was calculated by dividing the MFI values of RA-treated treated samples with the MFI values of respective controls. In case where the MFI of treated sample was less than the MFI of control sample, we calculated the fold decrease instead of calculating the fractional fold increase. Fold increase and decrease were labeled with “+”and “-”respectively. Statistical validation of the results was performed using two-tailed paired t-test on log-transformed (log-base 2) values of fold changes. P-values 0.05 and less were considered significant.

## Results

### RA treatment induces concomitant expression of meso- and endodermal lineage markers in hESC

The aim of this study was to analyze the potential changes in laminin expression in hESC during early steps of differentiation. To induce the differentiation of hESC, we replaced the normal growth medium (mTeSR1) with the differentiation medium containing 10 μM RA dissolved in DMSO (see Materials and Methods).

**Fig 1:**
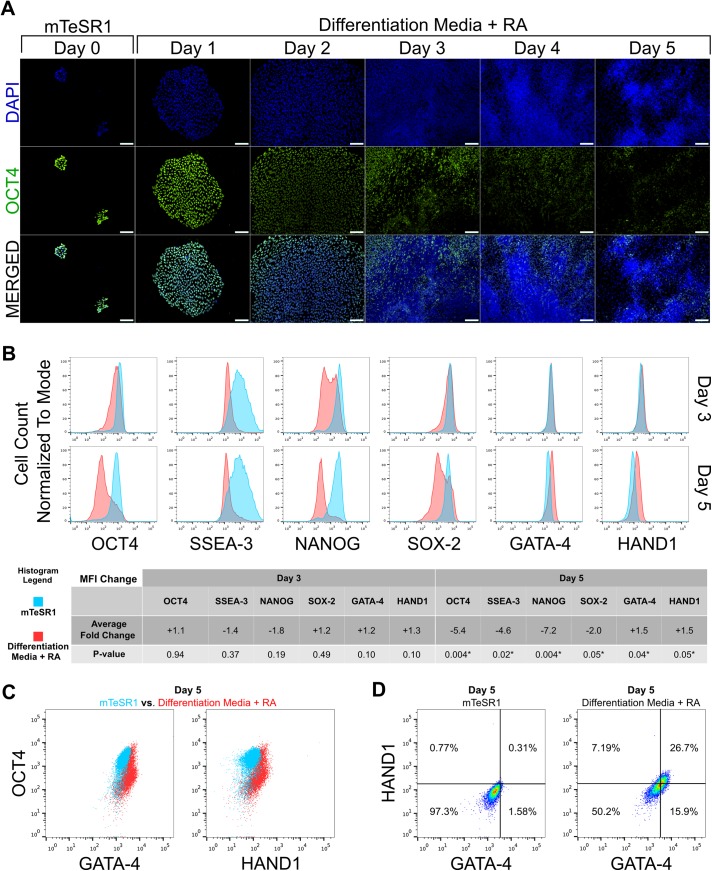
RA induces hESC differentiation and concomitant upregulation of HAND1 and GATA-4 expression. (A) Immunofluorescence analysis of OCT4 (green) in RA-treated hESC. Cell nuclei were labeled with DAPI (blue). Scale bar: 100 μm. (B) Flow cytometric analysis of OCT4, SSEA-3, NANOG, SOX-2, GATA-4 and HAND1 expression in RA-treated hESC on day 3 and 5. Untreated hESC (grown in mTeSR1) harvested at the identical time-points were used as controls. Average Fold Change based on Median Fluorescence Intensity (MFI) values was calculated in relation to corresponding control (mTeSR1) samples. Statistical significance with P-values less than 0.05 are labeled with “*” (C) Flow cytometric analysis of RA-treated and control (mTeSR1) hESC cells co-stained with antibodies recognizing OCT4 and GATA-4 or HAND1. Overlays of RA-treated (red) and control (mTeSR1, blue) hESC populations are presented. (D) Flow cytometric analysis of RA-treated and control (mTeSR1) hESC co-stained with GATA-4 and HAND1-specific antibodies. The percentages of cell populations in each quadrant are indicated on the density plots.

Cells grown under the different culture conditions showed distinctive morphology of colonies (S1A Fig). Although spontaneous differentiation of some cells was detected under normal culture conditions and in DMSO-containing media on day 5, the substantial changes were detected in RA-treated hESC colony morphology, which were characteristic to differentiating hESC (S1A Fig). The decrease in the expression of the pluripotency marker OCT4 in the centers of large colonies became apparent by day 2 and spread towards the edges of the colonies during the course of the experiment (Fig 1A). In addition to OCT4 we noted concomitant gradual down-regulation of pluripotency markers SOX2, NANOG, SSEA3 during the RA treatment of hESC as detected by flow cytometric analysis (Fig 1B). These results were confirmed by Western blot analysis of the RA-treated cells, which showed that overall expression of OCT4 remained high on day 3 but was decreased notably on day 5 (S1B Fig).

To characterize the RA-treated hESC in respect of their lineage commitment the cells were stained with antibodies specific to markers of ectoderm (SOX-1, OTX2), mesoderm (Brachyury, HAND1), endoderm (GATA-4, SOX-17), and extraembryonic tissues (CDX-2). At day 3 of RA treatment significant changes in several differentiation markers (OTX2, SOX-1, Brachyury, SOX-17 and CDX-2) were seen (S2 Fig). Since no significant changes in the expression of pluripotency markers could be detected at this time point the induction of expression of markers of all three germ layers and extraembryonic tissue can be considered nondefinitive. By day 5 of RA treatment drastic downregulation in the expression of pluripotency markers OCT4, NANOG, SOX-2 and SSEA-3 could be seen. Concomitantly, a significant increase in the expression of endodermal markers GATA-4 and SOX-17 and the mesodermal marker HAND1 were present in RA-treated hESC (Fig 1B, S2 Fig). GATA-4 and HAND1, which were present at the highest level in RA-treated hESC were preferentially expressed in hESC with low OCT4 content (Fig 1C). Furthermore, co-staining of RA-treated and control hESC with antibodies recognizing GATA-4 and HAND1 showed the shift of the whole RA-treated hESC cell population towards GATA-4 and HAND1 co-expression (Fig 1D). This shows that, in our hands, the 5-day treatment with RA induced downregulation of pluripotency markers and co-induction of mesodermal and endodermal lineage markers suggesting the commitment of hESC towards mesendodermal differentiation program [29].

### Quantitative and qualitative changes in laminin-511 and -521 expression during early hESC differentiation induced by RA

**Fig 2:**
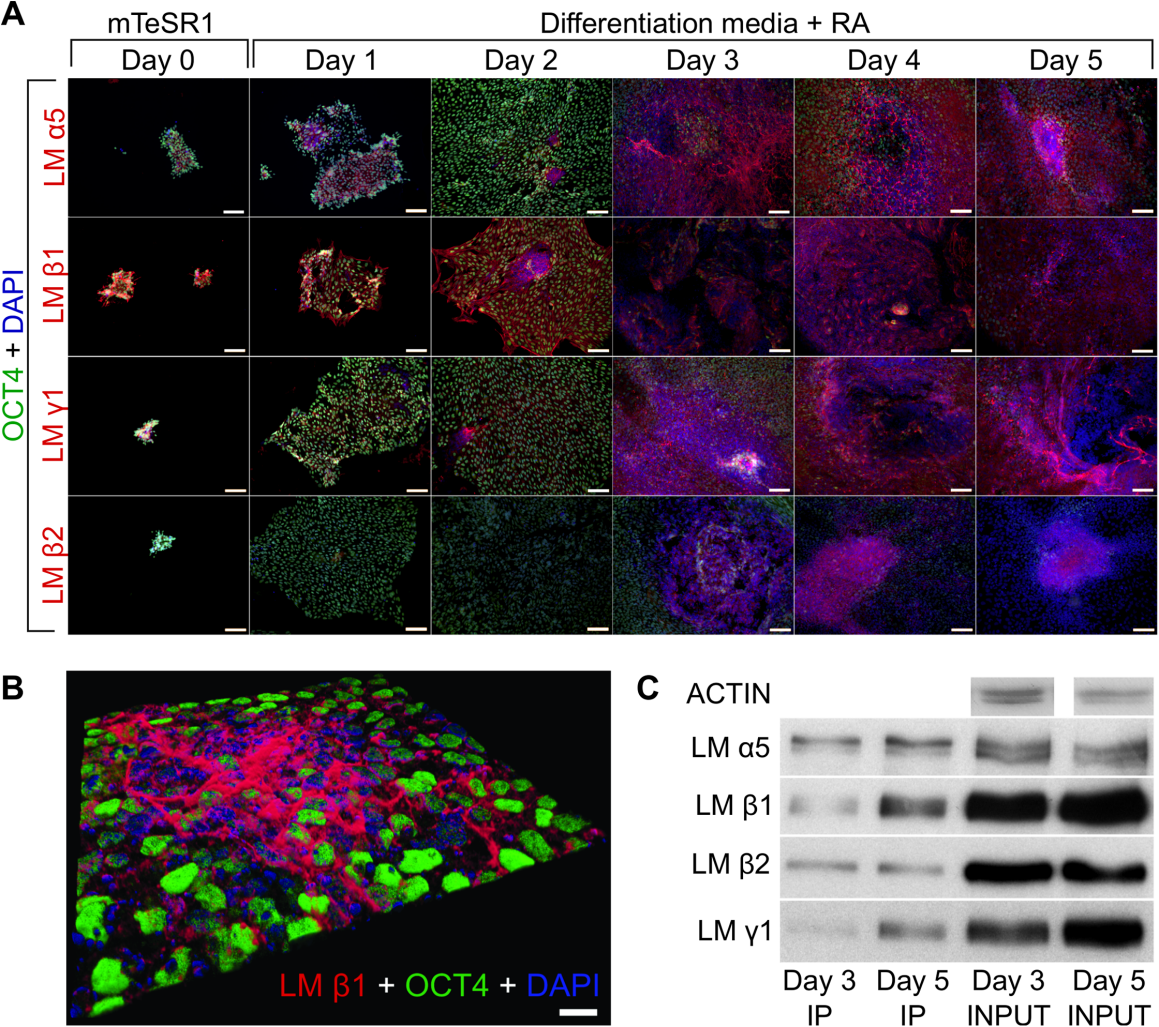
The expression of laminin α5, β1, β2 and γ1 chains in differentiating RA-treated hESC. (A) Immunofluorescence analysis of laminin (LM) chains α5, β1, β2 and γ1 and OCT4 in RA-treated hESC. Laminin chains (red) and OCT4 (green) were detected with appropriate antibodies. Cell nuclei were labeled with DAPI (blue). Scale bar: 100 μm. (B) Multilayer confocal microscopy was used to visualize the LM β1 chain (red) localization and OCT4 (green) expression in RA-treated hESC. Cell nuclei were labeled with DAPI (blue). Scale bar: 20 μm. (C) Immunoprecipitation of laminin-511 and -521 from RA-treated hESC. The protein complexes were immunoprecipitated using laminin α5 chain-specific antibody. The laminin α5, β1, β2 and γ1 chains were detected by Western blot analysis using corresponding antibodies.

It has been shown previously that hESC express laminin α5, β1, β2 and γ1 chains, which are the components of laminin-511 and -521 trimers [22]. To compare the expression pattern of these laminin chains in undifferentiated (day 0) and in RA-treated hESC (from day 1 to day 5), we performed stainings with antibodies recognizing laminin α5, β1, β2 and γ1 chains. To distinguish between pluripotent and differentiated cells, the samples were co-stained with an antibody recognizing the pluripotency marker OCT4. Gradual changes in the expression pattern of studied laminin chains were recognized during the early hESC differentiation (Fig 2). From day 0 to day 2 the colonies consisted mainly of OCT4-expressing undifferentiated cells and showed low and homogenous expression of laminin chains (Fig 2A). As an exception, high level of laminin β1 chain was detected already on day 0. Laminin α5 chain was detected in the center of larger colonies on day 1. From day 3 until day 5 of differentiation small areas with enhanced expression of laminin chains appeared in colony centers, were OCT4 was not detectable pointing at the differentiated cells (Fig 2A). To study the laminin expression pattern in detail, we analyzed laminin β1 chain expression by confocal microscopy in the hESC colonies treated with RA for 5 days. The staining with anti-laminin β1 chain antibody revealed a network of large fibers, which was located on top of the cells (Fig 2B). We confirmed that the laminin-rich ECM network coincided with differentiating hESC with downregulated OCT4 expression. At the same time the ECM in the vicinity of undifferentiated cells expressing high level of OCT4 contained less laminin β1.

To study the relative expression levels of trimeric laminin isoforms, we prepared protein extracts from RA-treated cells collected on day 3 and day 5 and immunoprecipitated (IP) laminin-511 and -521 using the laminin α5 chain specific antibody. The results showed an increased expression of laminin α5 in differentiating hESC (Fig 2C, Table B in S1 File). Furthermore, an increase in the amounts of β1 and γ1 chains bound to the α5 chain on day 5 of RA treatment when compared to that of day 3 could be detected while the amount of α5-bound β2 chain was not increased (Fig 2C, Table B in S1 File). This suggests that the relative amount of laminin-511 was increased during early steps of RA-induced differentiation and the relative amount of laminin-521 represented by laminin β2 chain was decreased.

To exclude the possibility that laminin α5 chain was associated with other laminin chains than β1, β2 and γ1 we incubated the IP blots with antibodies recognizing laminin β3, γ2 and γ3 chains (S3 Fig). No additional laminin isoforms containing laminin α5 chain were found.

### Laminin α5 chain expression is increased in the differentiating hESC

**Fig 3:**
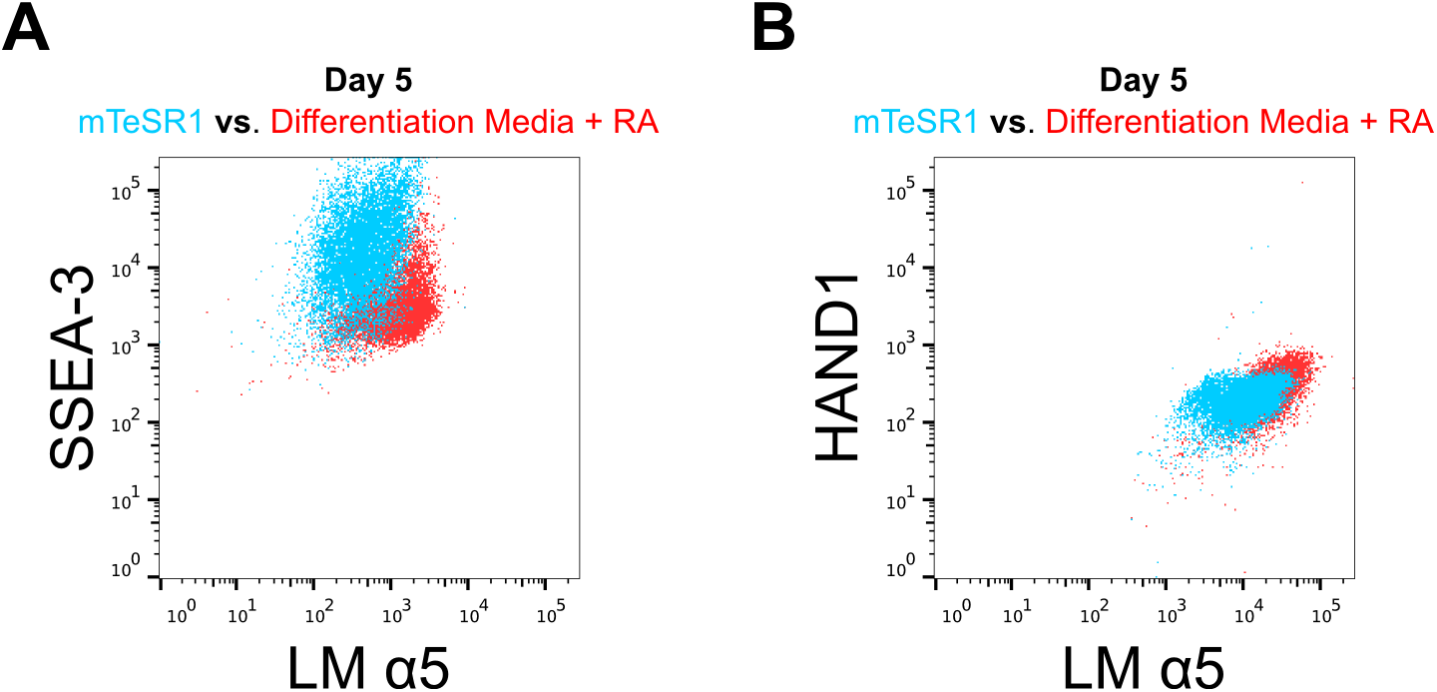
Laminin α5 chain expression increases in differentiating hESC. Flow cytometric analysis of RA-treated (red population) and control (mTeSR1, blue population) hESC stained with antibodies recognizing laminin (LM) α5 chain and SSEA-3 (A) or HAND1 (B).

Since our previous experiments showed an increased level of laminin α5 chain-containing laminins in RA-treated hESC culture lysates, we asked whether laminin α5 chain expression was specifically increased in differentiating hESC at the single cell level. First, we used antibodies specific to the pluripotency marker SSEA3 and laminin α5 chain to co-stain the hESC treated with RA for 5 days. Flow cytometric analysis showed that cells expressing SSEA-3 at low level contained more α5 laminin chain than those, which expressed high level of SSEA3 (Fig 3A). This finding led us to hypothesize that the committed hESC have a higher laminin α5 chain expression level. Indeed, when we co-stained the RA-treated hESC with antibodies recognizing HAND1 and α5 laminin chain we found that the cells with higher HAND1 expression showed also a higher expression of α5 laminin chain (Fig 3B).

### The expression pattern of laminin chains in undifferentiated and differentiating hESC

**Fig 4:**
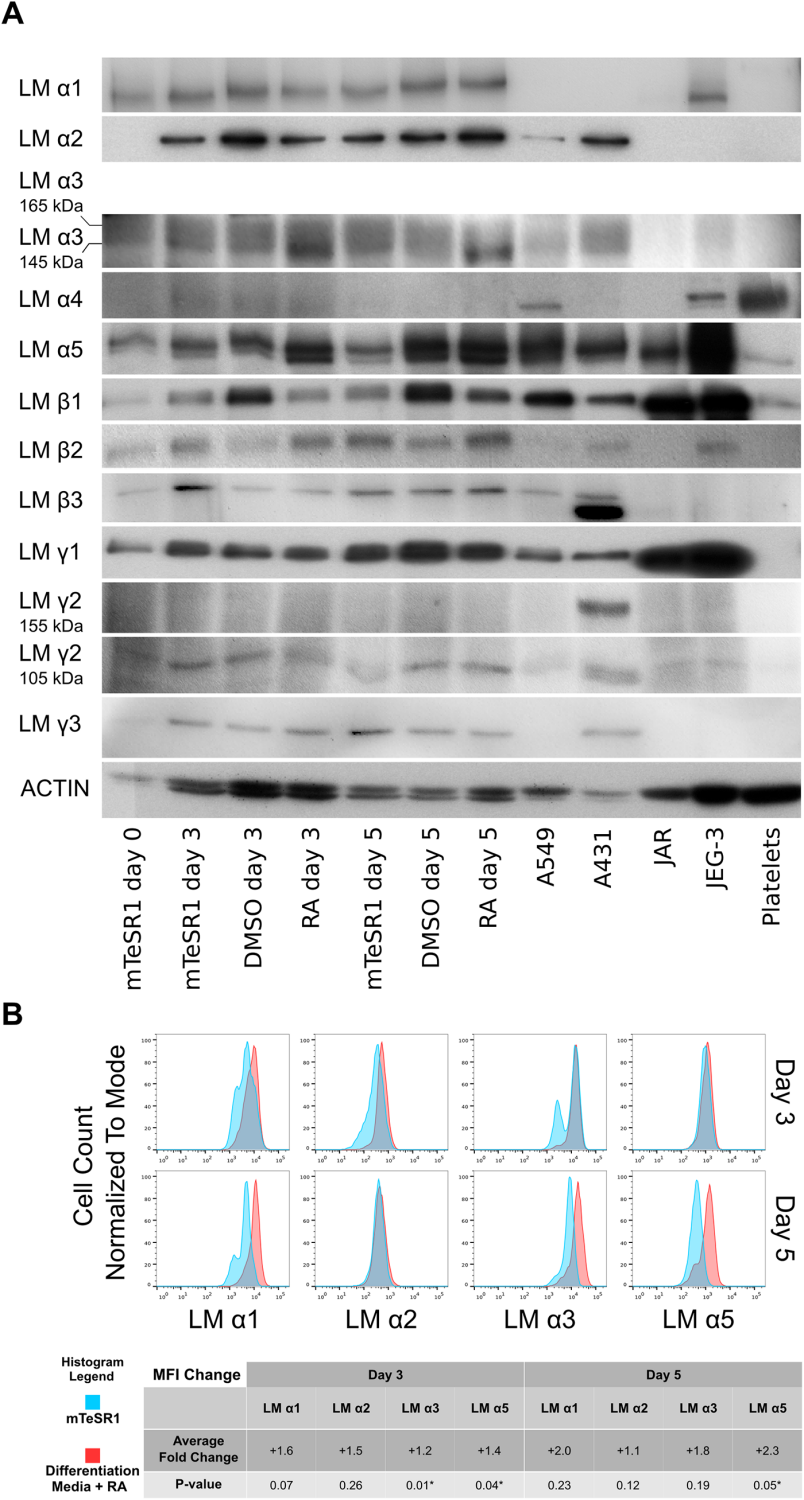
hESC express a diverse range of laminin chains. (A) Western blot analysis of indicated laminin (LM) chains in hESC. The lysates of JEG-3 (α1 chain), A431 (α2, α3, β1-β3, γ1- γ3), A549 and JAR (α5) cells and human platelets (α4) were used as controls. See the text for a detailed explanation. (B) Flow cytometric analysis of laminin (LM) chains α1, α2, α3 and α5 in hESC on day 3 and 5 of RA treatment. Untreated hESC (mTeSR1) were used as controls. Average Fold Change based on Median Fluorescence Intensity (MFI) values was calculated in relation to corresponding control (mTeSR1) samples. Statistical significance with P-values less than 0.05 are labeled with “*”.

Several groups have studied laminin expression pattern of undifferentiated hESC culture [21–24]. However, the researchers have focused on the laminin chain mRNA expression and the data regarding the expression of corresponding proteins is scarce. Furthermore, to our knowledge, there exist no published data describing the changes in laminin expression during early differentiation of hESC. Therefore, we aimed to describe the changes in laminin chain expression pattern at protein and mRNA level in qualitative terms during early differentiation of hESC induced by RA. We performed a comprehensive Western blot and RT-PCR analysis of RA-treated hESC samples collected at day 0, 3 and 5 using antibodies and PCR primers, which recognized laminin α1-α5, β1-β3 and γ1- γ3 chains. The hESC grown in mTeSR1 medium and in differentiation medium without RA (but including RA solvent DMSO) were used as controls (Fig 4, S4 Fig). The lysates from a selection of human tumor cell lines (A431, A549, JAR and JEG-3) and normal human platelets were used as positive controls for laminin chain expression [30–33] (see Materials and Methods).

We detected the presence of protein and mRNA of α1, α5, β1, β2 and γ1 chains in undifferentiated hESC as previously reported by others [21,22] as well as in in differentiating hESC (Fig 4A, S4 Fig). Additionally the presence of laminin α2, α3, β3, γ2, and γ3 chains in the hESC lysates was detected (Fig 4A), which have not been reported in hESC before at protein level. RT-PCR analysis showed the presence of all mRNAs corresponding to the detected laminin chains confirming the hESC as the origin of these proteins (S4 Fig).

Interestingly, specific variants of laminin α3, β3 and γ2 chains were detected in hESC (Fig 4A). Two variants of the laminin α3 chains (165 kDa and 145 kDa) [34] were detected in the hESC lysates. Although we aimed to show the expression of different laminin chains only at qualitative level, we noted that the 165 kDa variant was almost equally present in all the studied samples, while the 145 kDa variant was upregulated in the RA-treated hESC (Fig 4A). These laminin α3 chain variants were also present in the A431 cell line, which has been shown to express laminin-332 [31]. Furthermore, in accordance with the published data, two variants of laminin β3 chain with dissimilar electrophoretic mobility were detected in A431 cell lysate [31]. In hESC only the β3 chain variant with higher molecular mass was detected (Fig 4A). Since we were able to detect the presence of laminin β3 chain mRNA, albeit at a very low level (S4 Fig), we concluded that this laminin chain is expressed in hESC. Only the 105 kDa variant of the laminin γ2 chain was present in all hESC samples regardless of the hESC differentiation status (Fig 4A) while the reference cell line A431 expressed both known variants of this laminin chain (105 kDa and 155 kDa) [34].

Laminin α4 chain was not detected in hESC in Western blot assay, although it was detected in the positive control sample prepared from human platelets (Fig 4A). Since the mRNA of this laminin chain was barely detectable (S4 Fig) we concluded that the laminin α4 chain protein is absent in hESC.

To study the changes in the expression level of detected laminin α chains we performed a flow cytometric analysis of undifferentiated and RA-treated hESC cells stained with the antibodies recognizing α1, α2, α3 and α5 chains. On day 3 we detected increased expression of α3 and α5 laminins chain expression in RA-treated cells (Fig 4B). Although the expression of laminin α1 chain was also increased in RA-treated hESC, this increase was insignificant. Further increase in laminin α5 chain was detected on day 5 during the hESC differentiation (Fig 4B). The increased levels of α3 and α1 laminin chains were also detected on day 5 but variation from one experiment to another rendered these measurements insignificant.

## Discussion

The fact that hESC interact actively with their surrounding ECM has attracted a fair amount of attention recently. In particular, the research conducted in this field has shown that both laminin-511 and -521, which are expressed by hESC, support their undifferentiated growth in long-term cultures [22,25]. In concordance with this a recent study suggests that the α5-containing laminins produced by the hESC are necessary for their self-renewal [35]. The studies conducted so far have, however, focused on the pluripotent hESC cultures, but the potential changes in laminin repertoire during the early steps of hESC differentiation have not been studied yet.

In this study we characterized the changes in the expression of laminin α5, β1, β2 and γ1 chains during early differentiation of hESC in closer detail. To induce hESC differentiation, we utilized a RA-mediated protocol. Since our aim was to study the changes in laminin expression, which accompany the exit of hESC from pluripotent state we followed the cells only until the expression of pluripotency markers was substantially decreased. Consequently, by the end of the experiments (day 5) the RA treatment induced modest upregulation of mesodermal lineage marker HAND1 and endodermal lineage markers SOX-17 and GATA-4 in hESCs suggesting the induction of differentiation towards mesendodermal lineage [29]. The induction of mesendodermal markers is somewhat surprising since RA-treatment has been shown to induce neuronal differentiation in hESC [8]. There may be several reasons behind this unexpected observation. In general the changes in the expression pattern of differentiation markers at such early differentiation stages are poorly described and thus the final lineage commitment may differ from the early marker expression. Furthermore, there are studies where RA induced hESC differentiation towards ectodermal and endodermal lineages [9] or ectodermal and mesodermal lineages [36] suggesting that hESC responses to RA treatment are context dependent. In concordance with the latter, RA has also been described as a factor that promotes multilineage differentiation and needs to be combined with other inductive signals in order to induce hESC differentiation towards neuronal lineages [37].

We noted that the accumulation of the studied laminin chains was accompanied with the differentiation status of the hESC. The increased expression of laminin chains was first noted in the centers of RA-treated hESC colonies, which coincided with the decrease in the expression of the pluripotency marker OCT4 in the hESC nuclei. Furthermore, in the centers of differentiating colonies laminin accumulated into a network-like structure that may indicate formation of higher-order laminin complexes since the formation of polymeric networks is a well-known property of laminins [38]. Such laminin-containing structure could be needed for proper differentiation of hESC. For example, in early embryonic differentiation specific laminin-containing structure—the Reichert's and embryonic basement membranes—are formed, which guide further differentiation of the embryo [19].

Since laminin-511 and -521 differ in their ability to support the hESC clonal survival [25], we were interested to find out, whether their relative amounts in hESC colonies change during early differentiation. Our finding that laminin-511 but not -521 preferentially accumulates during early differentiation induced by RA suggests that laminin-511 is an important factor for the initiation or coordination of differentiation, while laminin-521 could be more associated with the hESC pluripotent state. To verify this hypothesis we used a laminin α5 chain-specific antibody (8G9), which preferentially blocks the binding of laminin-511 to cellular integrins [39]. However, the blocking experiments did reveal ambiguous effect of the blocking antibody on differentiation of hESC in the presence of RA (data not shown). Recently, α5-containing laminins were shown to be necessary for hESC self-renewal [35]. However, the potential functional differences between laminins 511 and 521 were not studied. Studies with mouse embryonic stem cells (mESC) have also suggested an important role for laminin-511 in the differentiation and showed that this particular laminin isoform promotes the mESC differentiation towards endodermal lineage [40,41]. Nevertheless, given the clear link between the induction of hESC differentiation and the increase in laminin-511 presented in current paper, its exact role in regulation of hESC differentiation status is yet to be elucidated in upcoming studies

Various groups have analyzed the expression pattern of laminin chains in hESC at mRNA level [21–24], while only a few publications have addressed this question at protein level [21,23]. The latter agree that the laminin α5, β1 and γ1 chains are expressed at detectable levels in hESC at protein level [21,23], which is in good concordance with our findings. Still, discrepancies exist in literature concerning the expression of other chains. Two studies show the presence of laminin α1 chain mRNA [21,22] and one study has demonstrated the presence of laminin α1 chain protein in hESC [23]. To our knowledge there exists only one study, which describes the presence of laminin α2 chain mRNA in hESC, however, the expression of α2 chain protein was not shown [22]. Two studies have detected the mRNA of laminin β2 chain in the hESC [21,22], while only one of them has additionally demonstrated the presence of the corresponding protein by immunofluorescence microscopy [21]. There exist other reports, however, which claim that laminin β2 chain mRNA [24] or corresponding protein [23] were not found in hESC. Furthermore, the researchers have detected the expression of γ2, γ3 and β3 chain mRNAs in hESC, nevertheless, the presence of corresponding proteins was not detected [21]. The expression of laminin chain γ3 was also detected by another group, however, the authors claim that the expression of α1, α2, β3, γ2 chain was not present in hESC [24].

In order to shed light on these discrepancies and to study the changes in laminin expression pattern at protein level during the induction of hESC differentiation, we conducted a comprehensive analysis of different laminin chains. Surprisingly, we were able to detect a number of laminin chains in cultured hESC. We confirmed the expression of previously identified laminin α1, α5, β1, β2 and γ1 chains at protein as well as at mRNA level in hESC. We showed for the first time the presence of laminin α2, α3, β3, γ2 and γ3 chains in hESC at protein levels. As the mRNA expression of laminin α2, β3, γ2 and γ3 chains has been shown before [21,22,24], the detection of relevant proteins in our study was not completely unexpected. In contrast, the presence of laminin α3 chain has not been previously detected in hESC [21–24]. Furthermore, we found that a specific variant of laminin α3 chain − 145 kDa − was upregulated in the RA-treated hESC. In concordance with previous studies, the laminin α4 chain was not detected in hESC [21–24]. According to our data, no major qualitative changes in the gross laminin chain expression pattern during the early steps of hESC differentiation were detected. Concomitantly, the mRNA analysis showed the presence of similar laminin chain pattern in every sample studied, confirming this conclusion.

One has to note that the differences between our and previously published data may arise from the use of specific hESC cell line and culture conditions. Furthermore, the findings of this study suggest that hESC may produce different laminins depending on their immediate surroundings. This adds an additional dimension to our understanding about the ability of hESC to modify and organize their microenvironment.

Our findings suggest that intricate alterations in laminin isoform balance rather than major qualitative changes take place during the early steps of hESC differentiation. As an example we demonstrate that laminin-511 but not -521 preferentially accumulates during early differentiation of hESC. Given that α5-containing laminin isoforms produced by hESC also contribute to the maintenance of their pluripotency [22,25], this suggests that changes in the balance of laminin-511 and laminin-521 isoform expression might guide hESC early differentiation. Further cell culture experiments utilizing different laminin-511 and -521 ratios in growth matrix could clarify the potential use of our findings in ex vivo hESC culture and differentiation protocols.

One has to keep in mind, however, that ECM is a complex network of proteins and the changes in ECM composition during hESC differentiation are likely to involve many other ECM components apart from laminins. The elucidation of these changes is a compelling topic for further investigations.

## Supporting Information

S1 Fig Cultivation in the presence of 10 μM RA induces differentiation of hESC. For the initiation of hESC differentiation, one day after passage (day 0) the mTeSR1 media was replaced with the differentiation media, which contained 10 μM RA or DMSO (control). (A) Changes in colony appearance during RA treatment of hESC. When compared with the cells grown in mTeSR1 media, the RA- or DMSO-treated hESC colonies are less homogeneous. The irregular shape of RA-treated hESC is a hallmark of differentiation. Scale bar: 100 μm. (B) Western blot analysis of OCT4 expression in undifferentiated (mTeSR1), mock-treated (DMSO) and RA-treated hESC harvested at indicated timepoints.
(TIF)

S2 Fig Expression of lineage-specific differentiation markers in RA-treated hESC. Flow cytometric analysis of OTX2, SOX-1 (ectodermal markers), Brachyury (mesodermal marker), SOX-17 (endodermal marker) and CDX-2 (extra-embryonal lineage marker) expression in hESC on day 3 and 5 of RA treatment. Untreated hESC (mTeSR1) harvested at the same timepoints were used as controls. Average Fold Change based on Median Fluorescence Intensity (MFI) values was calculated in relation to corresponding control (mTeSR1) samples. Statistical significance with P-values less than 0.05 are marked with “*”.
(TIF)

S3 Fig Laminin α5 chain is not associated with the β3, γ2 and γ3 chains in RA-treated hESC. Immunoprecipitation was performed with laminin α5 chain-specific monoclonal antibody. The laminin (LM) β3, γ2 and γ3 chains were detected by Western blot analysis using chain-specific antibodies.
(TIF)

S4 Fig The expression pattern of laminin chain mRNAs in hESC. RT-PCR analysis of total RNA isolated from hESC grown in differentiating media with or without RA and in mTeSR1 (control cells). Primer sets used for the detection of different laminin chains are described in Supporting Information (Table C in S1 File).
(TIF)

S1 File The list of antibodies and primers used in the study and summarized results of quantification of immunoprecipitated material. (DOC)
