# Supplementary figures and images for: Changes in Laminin Expression Pattern during Early Differentiation of Human Embryonic Stem Cells
Source: PLoS One. 2015 Sep 17;10(9):e0138346. doi: 10.1371/journal.pone.0138346 (PMC4574950; doi:10.1371/journal.pone.0138346)

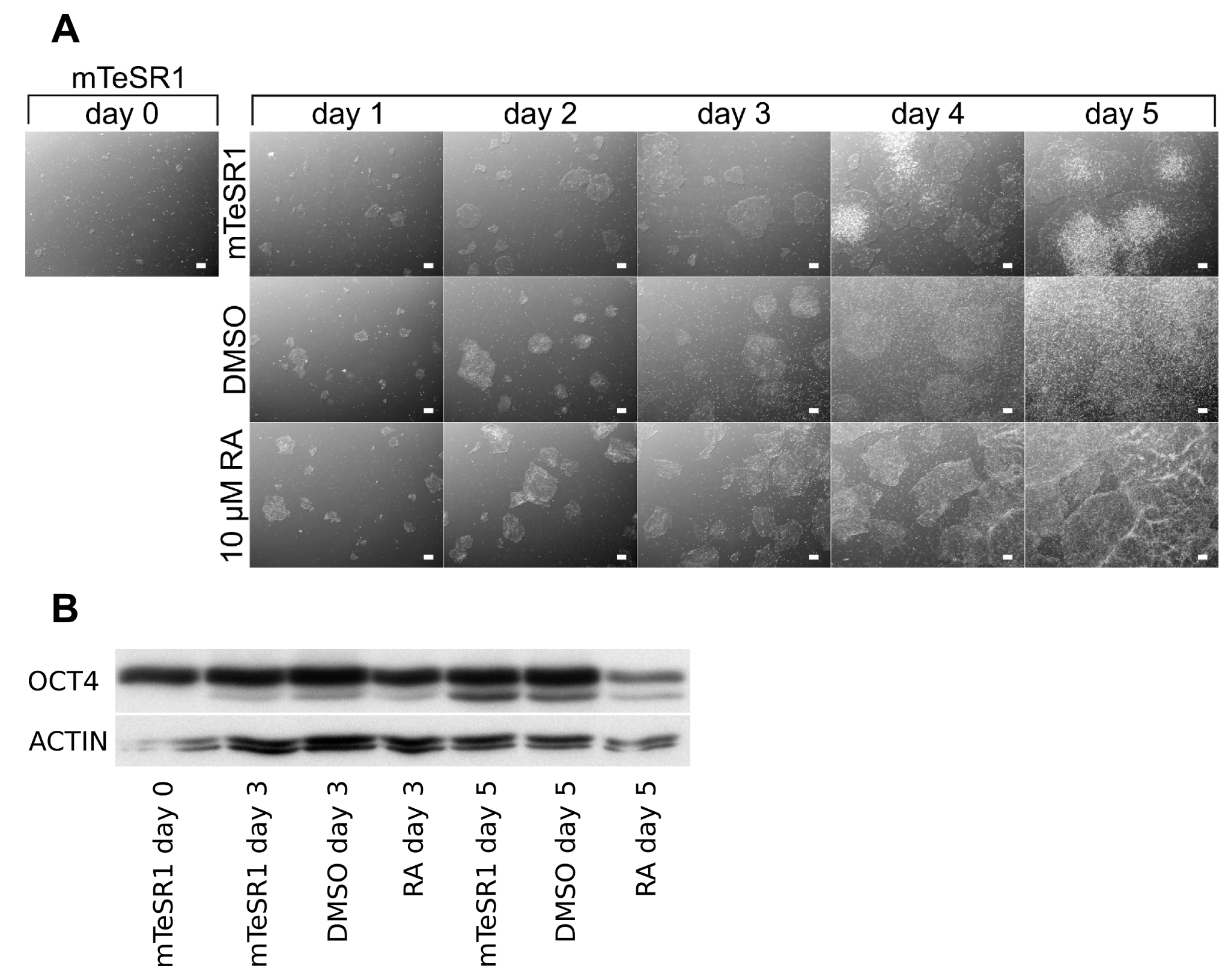

Supplement: S1 Fig — For the initiation of hESC differentiation, one day after passage (day 0) the mTeSR1 media was replaced with the differentiation media, which contained 10 μM RA or DMSO (control). (A) Changes in colony appearance during RA treatment of hESC. When compared with the cells grown in mTeSR1 media, the RA- or DMSO-treated hESC colonies are less homogeneous. The irregular shape of RA-treated hESC is a hallmark of differentiation. Scale bar: 100 μm. (B) Western blot analysis of OCT4 expression in undifferentiated (mTeSR1), mock-treated (DMSO) and RA-treated hESC harvested at indicated timepoints. (TIF) [file pone.0138346.s001.tif]

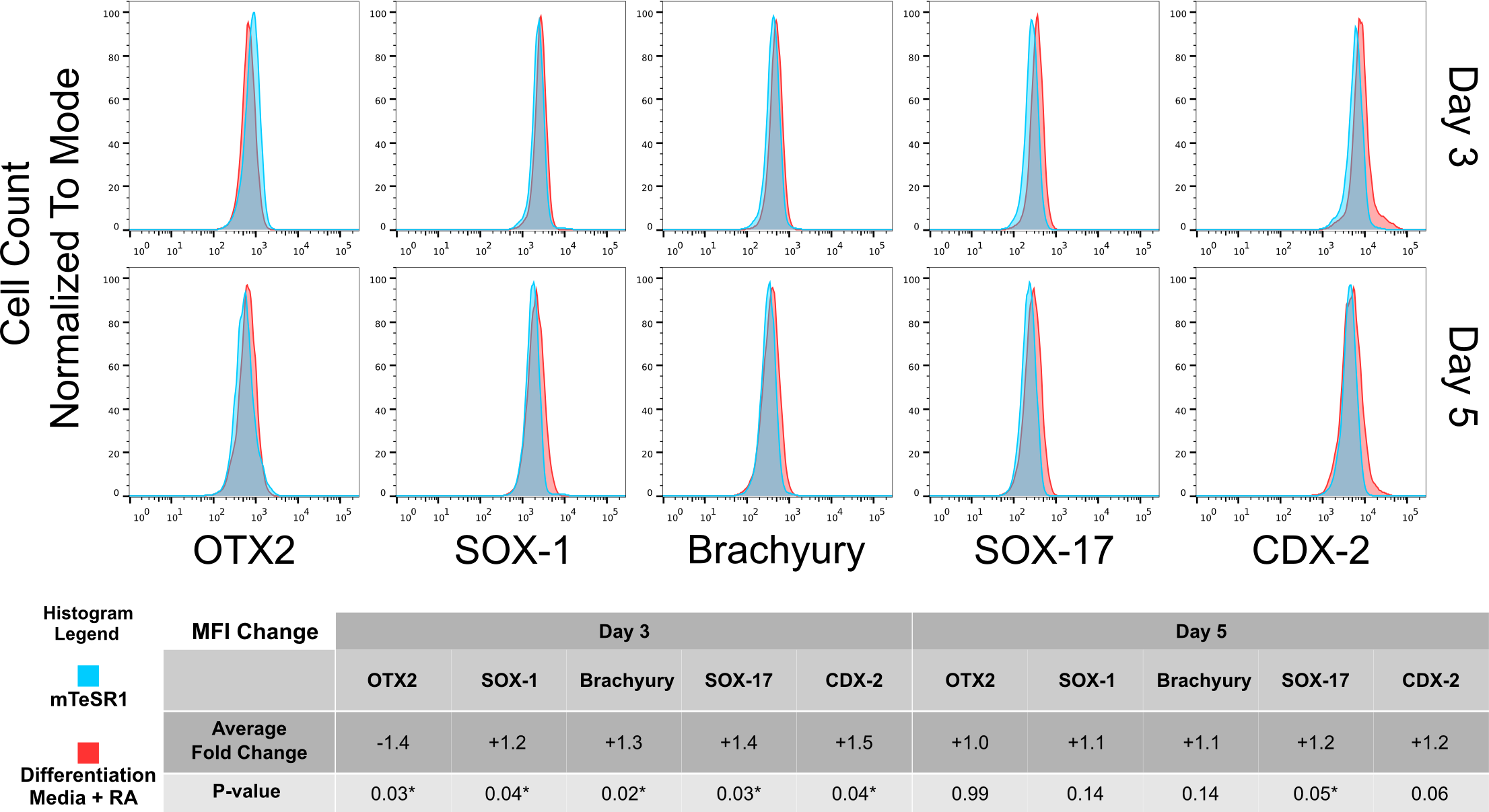

Supplement: S2 Fig — Flow cytometric analysis of OTX2, SOX-1 (ectodermal markers), Brachyury (mesodermal marker), SOX-17 (endodermal marker) and CDX-2 (extra-embryonal lineage marker) expression in hESC on day 3 and 5 of RA treatment. Untreated hESC (mTeSR1) harvested at the same timepoints were used as controls. Average Fold Change based on Median Fluorescence Intensity (MFI) values was calculated in relation to corresponding control (mTeSR1) samples. Statistical significance with P-values less than 0.05 are marked with “*”. (TIF) [file pone.0138346.s002.tif]

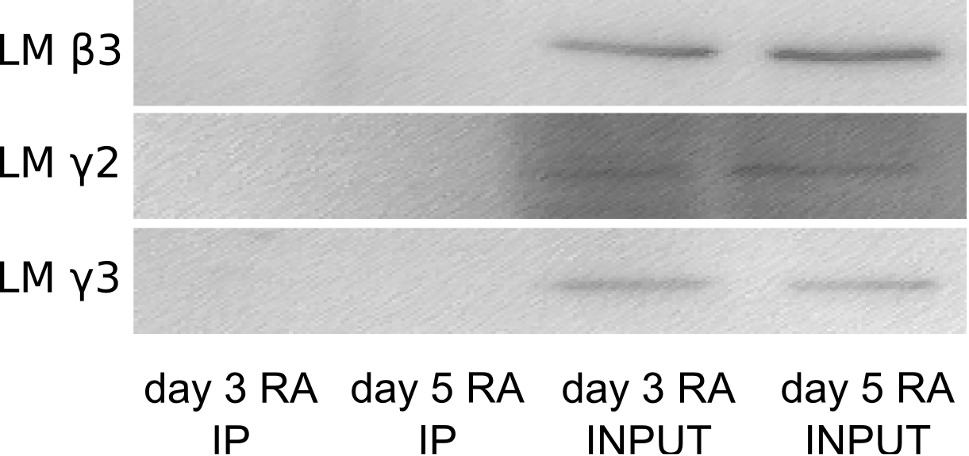

Supplement: S3 Fig — Immunoprecipitation was performed with laminin α5 chain-specific monoclonal antibody. The laminin (LM) β3, γ2 and γ3 chains were detected by Western blot analysis using chain-specific antibodies. (TIF) [file pone.0138346.s003.tif]

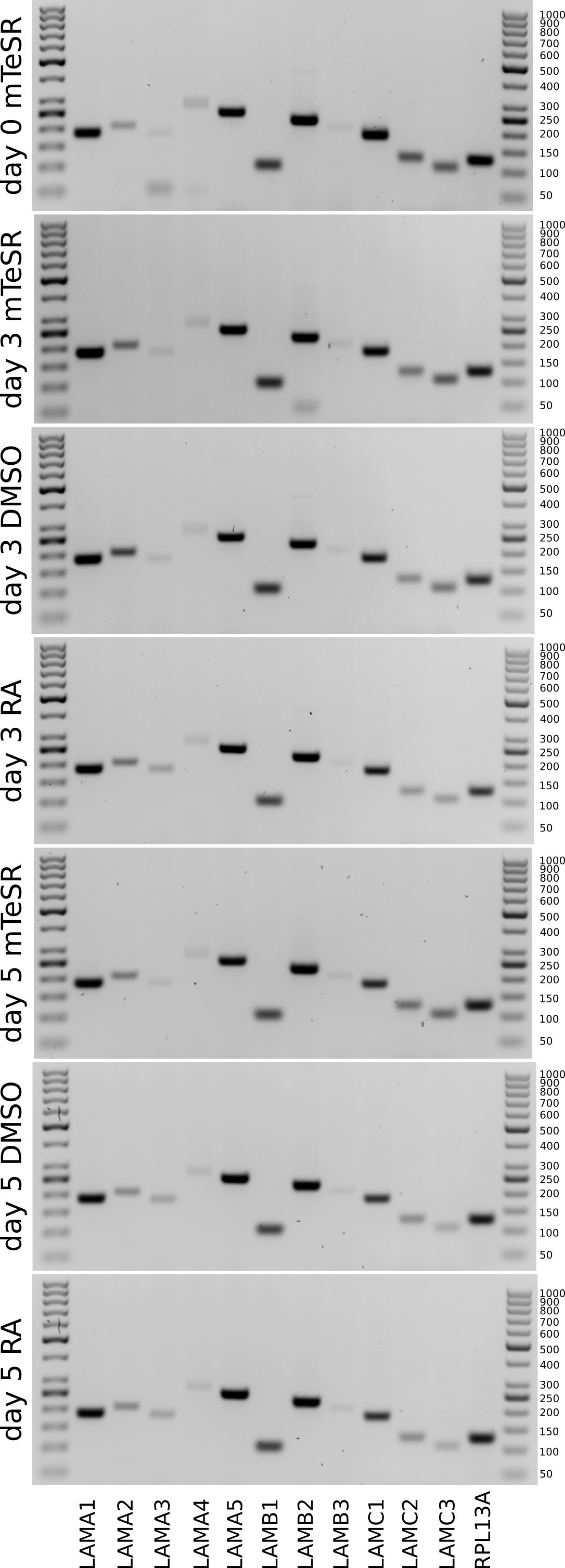

Supplement: S4 Fig — RT-PCR analysis of total RNA isolated from hESC grown in differentiating media with or without RA and in mTeSR1 (control cells). Primer sets used for the detection of different laminin chains are described in Supporting Information (Table C in S1 File). (TIF) [file pone.0138346.s004.tif]
