# Supplementary material for: Changes in Laminin Expression Pattern during Early Differentiation of Human Embryonic Stem Cells
Source: PLoS One. 2015 Sep 17;10(9):e0138346. doi: 10.1371/journal.pone.0138346 (PMC4574950; doi:10.1371/journal.pone.0138346)
Supplement: S1 File — (DOC) [file pone.0138346.s005.doc]

**Table A. Antibodies used in the study. WB- western blot; IF- immunofluorescence; BE - blocking experiment FC-flow cytometry assay**

| **Specificity** | **Name** | **Application** | **Host and clonality** | **Supplier** |
| --- | --- | --- | --- | --- |
| Laminin α1 | sc-5582 (H-300) | WB, FC | Rabbit polyclonal | Santa Cruz Biotechnology |
| Laminin α2 | sc-55605 (B-4) | WB, FC | Mouse monoclonal IgG1 | Santa Cruz Biotechnology |
| Laminin α3 | sc-20143 (H-187) | WB, FC | Rabbit polyclonal | Santa Cruz Biotechnology |
| Laminin α4 | 8C10 | WB | Mouse monoclonal IgG | S. Ingerpuu (Wondimu et al., 2004) |
| Laminin α4 | 3H2 | WB | Mouse monoclonal IgG | S. Ingerpuu (Wondimu et al., 2004) |
| Laminin α5 | 4B5 | WB, BE | Mouse monoclonal IgG | S. Ingerpuu (Wondimu et al., 2013) |
| Laminin α5 | 8G9 | BE | Mouse monoclonal IgG | S. Ingerpuu (Wondimu et al., 2013) |
| Laminin α5 | 4B12 | IF, FC | Mouse monoclonal IgG | S. Ingerpuu (Pook et al., 2013) |
| Laminin β1 | DG10 | WB, IF | Mouse monoclonal IgG1 | I. Virtanen |
| Laminin β2 | sc-59980 (C-4) | WB | Mouse monoclonal IgG1 | Santa Cruz Biotechnology |
| Laminin β3 | sc-20775 (H-300) | WB | Rabbit polyclonal | Santa Cruz Biotechnology |
| Laminin γ1 | sc-17751 (D-3) | WB | Mouse monoclonal IgG1 | Santa Cruz Biotechnology |
| Laminin γ1 | MAB1920 (2E8) | IF | Mouse monoclonal IgG1 | Millipore |
| Laminin γ2 | sc-20776 (H-183) | WB | Rabbit polyclonal | Santa Cruz Biotechnology |
| Laminin γ3 | sc-16601 (C19) | WB | Goat polyclonal | Santa Cruz Biotechnology |
| OCT4 | C30A3 | IF | Rabbit polyclonal | Cell Signaling Technology |
| OCT4 | sc-5279 (C-10) | WB | Mouse monoclonal IgG2b | Santa Cruz Biotechnology |
| OCT4 (Alexa-647-conjugate) | 560329 (40/Oct3) | FC | Mouse monoclonal IgG1 | BD Biosciences |
| OCT4 (PerCp-Cy5.5-conjugate) | 560794 (40/Oct3) | FC | Mouse monoclonal IgG1 | BD Biosciences |
| SSEA3 (Alexa-488-conjugate) | 53-8833 (MC-631) | FC | Rat monoclonal IgM | eBioscience |
| NANOG (PE-conjugate) | 560483 (N31-355) | FC | Mouse monoclonal IgG1 | BD Biosciences |
| SOX-2 (PerCp-Cy5.5-conjugate) | 561506 (030-678) | FC | Mouse monoclonal IgG1 | BD Biosciences |
| SOX-1 (NL-493-conjugate) | From SC022 KIT | FC | Goat polyclonal | R&D Systems |
| Brachyury (NL-557-conjugate) | From SC022 KIT | FC | Goat polyclonal | R&D Systems |
| SOX-17 (NL-637-conjugate) | From SC022 KIT | FC | Goat polyclonal | R&D Systems |
| OTX2 (NL-557-conjugate) | From SC022 KIT | FC | Goat polyclonal | R&D Systems |
| HAND1 (NL-637-conjugate) | From SC022 KIT | FC | Goat polyclonal | R&D Systems |
| GATA-4 (NL-493-conjugate) | From SC022 KIT | FC | Goat polyclonal | R&D Systems |
| CDX-2 (Alexa-488-conjugate) | ab195007 [EPR2764Y] | FC | Rabbit monoclonal | Abcam |
| Isotype control (Alexa-488-conjugate) | 53-4341-80 | FC | Rat IgM | eBioscience |
| Isotype control (PE-conjugate) | 554680 | FC | Mouse IgG1 | BD Biosciences |
| Isotype control (Alexa-647-conjugate) | 557783 | FC | Mouse IgG1 | BD Biosciences |
| Isotype control (PerCp-Cy5.5-conjugate) | 552834 | FC | Mouse IgG1 | BD Biosciences |
| Mouse IgG control | sc-2025 | IF, FC | Mouse IgG | Santa Cruz Biotechnology |
| Rabbit IgG control | ab27478 | IF, FC | Rabbit polyclonal | Abcam |
| Actin | sc-1616-R (I-19) | WB | Rabbit polyclonal | Santa Cruz Biotechnology |
| Anti-goat (HRP-conjugate) | sc-2020 | WB | Donkey polyclonal | Santa Cruz Biotechnology |
| Anti-mouse (HRP-conjugate) | 31430 | WB | Goat polyclonal | Thermo Scientific |
| Anti-rabbit (HRP-conjugate) | 7074 | WB | Goat polyclonal | Cell Signaling Technology |
| Anti-mouse (Alexa-647-conjugate) | A21237 | IF, FC | Goat polyclonal, F(ab')2 | Invitrogen |
| Anti-rabbit (Alexa-488-conjugate) | A11034 | IF, FC | Goat polyclonal | Invitrogen |

**Table B. Summarized results of quantification of immunoprecipitated material.** Quantification values were normalized to the values of Actin or LM α5 as indicated. Fold Change is calculated between quantification values from day 3 and day 5 of RA-treated hESC samples analyzed by Western blot.

|  | **Experiment I** | **Experiment II** |  |
| --- | --- | --- | --- |
|  | **Fold Change day3/day5** | **Fold Change day3/day5** | **Average Fold Change** |
| **LM α5/Actin** | 4.44 | 1.37 | 2.90 |
| **LM β1/LM α5** | 1.86 | 1.15 | 1.51 |
| **LM β2/LM α5** | -1.58 | -1.03 | -1.25 |
| **LM γ1/LM α5** | 1.17 | 1.88 | 1,53 |

**Table C. Primers used in RT-PCR.**

| **Gene name** | **Forward primer** | **Reverse primer** | **Product size** |
| --- | --- | --- | --- |
| LAMA1 | GTCAGCGACTCAGAGTGTTTG | AACTTGGGTGAAAGATCGTCAG | 185 bp |
| LAMA2 | GAACCCGCAGTGTCGAATCT | GGGGAGTTAGCTGCCTTCA | 204 bp |
| LAMA3 | TAGACTTTGGAAGCACCTACTCA | GTTTATCAAGGACACCACAACCT | 182 bp |
| LAMA4 | GCAGTGGAAATTCAGATCCCA | TAACCGCAGGTCATCAGTCAG | 293 bp / 254 bp / 272 bp |
| LAMA5 | GGTGTGTCTCTGCGTGACAA | CCCCGACGTAGAAGACGAA | 253 bp |
| LAMB1 | AGGAACCCGAGTTCAGCTAC | CACGTCGAGGTCACCGAAA | 103 bp |
| LAMB2 | GCCCTGGGAACTTCGACTG | GGAAGCACTTCTTTTCGTCCTG | 227 bp |
| LAMB3 | TCCTCTTGTGTTTTGCCCTG | CTGCCTGGAGTCACACTTG | 206 bp |
| LAMC1 | TCGTCAACGCCGCTTTCAA | GTGTCGGCCTGGTTGTTGTA | 184 bp |
| LAMC2 | CCAGGAGGGAAGTCTGTGATT | GCAGTGAATCCCATCAGTGTT | 128 bp |
| LAMC3 | CCAGGTGCATCACATCCTGAG | GACCCCATTTGGGCTCCATT | 106 bp |
| RPL13A | CCTGGAGGAGAAGAGGAAAGAGA | TTGAGGACCTCTGTGTATTTGTCAA | 126 bp |
| POU5F1 | CTGGAGCAAAACCCGGAGG | CCTCAAAGCGGCAGATGGTC | 181 bp |
| TUBB3 | CTCAGGGGCCTTTGGACATC | CAGGCAGTCGCAGTTTTCAC | 160 bp |

The primers for detecting laminin chains were the same as described in the reference [1].

RT-PCR program: 1 cycle (95^o^ C 5 min); 28 cycles (95^o^ C 30 sek, 58^o^ C 30 sek, 72^o^ C 30 sek); 1 cycle (72^o^ C 10 min)

References:

Evseenko D, K Schenke-Layland, G Dravid, Y Zhu, Q-L Hao, J Scholes, XC Wang, WR Maclellan and GM Crooks (2009). Identification of the critical extracellular matrix proteins that promote human embryonic stem cell assembly. Stem Cells Dev 18: 919–928.
